# Modeling Sexual Differences of Body Size Variation in Ground Beetles in Geographical Gradients: A Case Study of *Pterostichus melanarius* (Illiger, 1798) (Coleoptera, Carabidae)

**DOI:** 10.3390/life12010112

**Published:** 2022-01-13

**Authors:** Sergey Luzyanin, Anatoly Saveliev, Nadezhda Ukhova, Iraida Vorobyova, Igor Solodovnikov, Anatoliy Anciferov, Rifgat Shagidullin, Teodora Teofilova, Sargylana Nogovitsyna, Viktor Brygadyrenko, Viktor Alexanov, Raisa Sukhodolskaya

**Affiliations:** 1 Department of Ecology and Environmental Science, Kemerovo State University, 650000 Kemerovo, Russia; sl_luzyanin@mail.ru; 2 Institute of Ecology and Environmental Science, Kazan (Volga Region) Federal University, 420000 Kazan, Russia; anatoly.saveliev.aka.saa@gmail.com; 3 Visim Nature Reserve, 624140 Kirovograd, Russia; ukh08@yandex.ru; 4 Department of Biology, Mariy State University, 424000 Yoshkar-Ola, Russia; vigir@mail.ru; 5 Department of Zoology and Botany, Vitebsk State University Named after P. M. Masherov, 210038 Vitebsk, Belarus; iasolodov@mail.ru; 6 Kostroma Museum-Reserve, 156000 Kostroma, Russia; ancifer.ost@yandex.ru; 7 Institute of Ecology and Mineral Resource Management, Academy of Sciences of Tatarstan Republic, Tatarstan, 420000 Kazan, Russia; Shagidullin_@mail.ru; 8 Institute of Biodiversity and Ecosystem Research, Bulgarian Academy of Sciences, 1000 Sofia, Bulgaria; oberon_zoo@abv.bg; 9 Institute for Biological Problems of Cryolithozone Siberian Branch Russian Academy of Sciences, 677980 Yakutsk, Russia; sarnogov@mail.ru; 10 Department of Zoology and Ecology, Dnipro State Agrarian and Economic University, 49600 Dnipro, Ukraine; brigad@ua.fm; 11 State Budgetary Institution of Kaluga Region “Parks Directorate”, 248000 Kaluga, Russia; victor_alex@list.ru

**Keywords:** body size variation, ground beetles, sexual size dimorphism, geographical gradients, regression analysis

## Abstract

The aim of this study was to test the steepness of body size variation in males and females in the widespread ground beetle *Pterostichus melanarius* in geographical gradients. Beetles were sampled in 15 regions of Europe and Asia, and sampling territories differed 17° in latitude and 121° in longitude. We measured six linear traits in every captured beetle and formed a data set that included 2154 individuals. Body size variation in all traits in general was sawtooth, both in latitude and in longitude gradients. Regression analysis showed slight trends: in the latitude gradient, elytra parameters increased, pronotum length did not change but the width increased, and head parameters decreased. In the longitude gradient, the changes were as follows: elytra length increased, but its width did not change; pronotum length did not change, but its width increased; the head parameters decreased. Thus, we observed the elytra length increase and the head parameters decrease northwards and eastwards. We compared female and male regression curves (trait size on latitude/longitude): p-levels were significant only in four cases out of 12. Thus, we conclude that, in general, there is no evidence for the steepness in trait variation in males compared with females.

## 1. Introduction

A fundamental problem for biologists is to discover the processes driving intra- and interspecific variation in phenotypic traits [1]. Body size is an essential trait to study, because size variation directly affects fitness, physiology, and most life-history traits [2,3]. It is one of the key life-history traits in ectotherms [4,5], because it is considered a proxy for fecundity, body condition, and survival [6,7,8]. In general, larger individuals show higher fecundity and lower mortality rates [9,10]. Furthermore, species with wide distributional ranges show clines in body size. These may be the result of “ecogeographical rules” that discuss spatial patterns of phenotypic variation in terms of environmental variation [11,12].

Ground beetles are an interesting group in which to test ecogeographical rules because some species are widely distributed in the world and their biology and ecology are well known. In addition, they are the recognized bioindicators and respond to environmental fluctuations. Broad-scale patterns of body size variation in carabids are described poorly and have been studied in a very narrow spectrum of species [13].

Bergmann’s rule is a common ecogeographical pattern. It was formulated initially for homeothermic vertebrates and predicts their body size increase in high latitudes, as larger individuals have a smaller surface area to volume ratio [14]. However, in poikilotherms, the rule cannot be implemented: in insects, 30% of 63 datasets reported converse Bergmann clines (body size decreases with latitude), and 41% were not consistent with either pattern [15].

Within Carabidae (Coleoptera), Bergmann’s rule has been previously investigated only for a few species, distributed predominantly in Russia [16,17]. Their body size variation was genus-biased: for *Carabus* Linnaeus, 1758 species decreased in size with latitude gradient, while for *Pterostichus* Bonelli, 1810 species had body size variation that was sawtooth, and *Poecilus cupreus* (Linnaeus, 1758) did not change at all. Several mechanisms can drive intraspecific variation in body size for ectotherms. The first is the temperature–size rule, which postulates larger body sizes at higher latitudes, because at lower ones, with higher temperatures, insects reach maturity faster and at a smaller size [18]. This mechanism is undoubtedly relevant to small bodied insects. However, there were a lot of exceptions in investigating body size variation when rearing temperatures differed, and this rule was confirmed in a limited number of species [19,20]. Ecological variation can also lead to broad ecogeographical patterns in body size, unconnected to temperature per se.

Latitude covaries with other ecological factors, including primary productivity, predation, competition, food abundance, desiccation resistance, and starvation resistance, which may be alternative drivers of biogeographical patterns [21,22,23,24,25]. For example, the resistance hypothesis proposes that larger organisms are more likely to resist the detrimental effects of starvation or desiccation, and therefore predicts that body size should increase with latitude and altitude [3,26]. Similarly, seasonality can favor converse Bergmann’s clines or a non-linear relationship between body size and latitude, as the limited time for foraging and growth at higher latitudes could potentially favor faster development and maturation at smaller sizes [27]. Variation in body size may be driven by a variety of mechanisms across divergent species; therefore, looking at ecogeographical patterns across species with similar physiology and behavior may be instructive for identifying the underlying causes of these patterns [28].

The problem of size variation in males and females has long been discussed in the literature. Some studies have presented higher amplitude in males size variation in geographical gradients [29]. However, particular investigations into ground beetle body size variation have not supported that opinion. On the contrary, some studies have shown that males are more variable neither in latitude nor in altitude gradients [30,31].

In our work, we hypothesized that (i) body size variation in the studied species, *Pterostichus melanarius* (Illiger, 1798) (Coleoptera, Carabidae), should be sawtooth because of the breeding peculiarities of that species; (ii) different traits vary differently in latitude/longitude gradients; (iii) trait variation in males should not be steeper than in females in the mentioned gradients.

## 2. Materials and Methods

### 2.1. Study Organism

*Pterostichus melanarius* is a very prolific and widespread European-Siberian beetle. Its southern distribution extends to northern Spain and its eastern—to the Amur River [32]. It was introduced in North America [33,34]. It is an eurytopic species occurring in open habitats (meadows, agricultural fields) and in all types of forests and gardens as well [35,36,37]. *Pterostichus melanarius* is a transpalearctic mesophilic zoophagous, generalist species [38,39,40,41]. It is an autumn breeder [38], with a polyvariant life cycle with multiseasonal reproduction and hibernation of larvae and adults of the first and second years of life [42,43].

Feeding resources affect the distribution of *P. malanarius* [44]. Its preferences in the selection of potential food objects have been studied: in a laboratory experiment, *P. melanarius* consumed Formicidae, Silphidae larvae, small species of Staphylinidae, Coccinellidae, Dermestidae, larvae of Lepidoptera, Hemiptera, Thomisidae, Opiliones and Lumbricidae [45,46]; this species readily feeds on other carabid species (*Stomis pumicatus* (Panzer, 1795), *Harpalus amplicollis* Mentries, 1848, *Panagaeus bipustulatus* (Fabricius, 1775), *Leistus ferrugineus* (Linnaeus, 1758), *Notiophilus laticollis* Chaudoir, 1850, species of *Bembidion* Latreille, 1802 and *Amara* Bonelli, 1810 genera, and other carabid’s larvae). In turn, other large predators (ground beetles *Carabus granulatus* Linnaeus, 1758, *Broscus cephalotes* (Linnaeus, 1758), and staphylinids, e.g., *Staphylinus caesareus* Cederhjelm, 1798) prey on *P. melanarius* [47].

As a generalist species, *P. melanarius* is regarded as a good model species for the purposes of studying the morphometric variation in differing environments [48]. Its high abundance in a wide range of biotopes (including islands and flooded plots on flood-plains and urbanized cenoses) has allowed vast databases on morphometric variation in this species to be created. The latter have been used in studies of body size variation in insects along geographical gradients and also in relation to carabids’ adaptation to anthropogenic impact [49,50].

Climate changes affect life cycles, and the future range of *P. melanarius* has been modeled accordingly [51]. Modeling processes have drawn prognostic maps of the species’ distribution related to climate change according to four scenarios. Mean annual temperatures and the mean temperature in the warmest and the coldest quarters of the year were the major factors affecting the spatial distribution of *P. melanarius*. Visualization of the potential range according to RCP 2.6, RCP 4.5, and RCP 6.0 scenarios predicted a range reduction by 2050, but its recovery by 2070. According to the RCP 8.5 scenario, the range of the species studied will be significantly transformed: by 2070, the range will shift towards the north-west in the continental European states but to the northeast in the coastal states. By 2070, almost all southern territories of Europe will become unsuitable for *P. melanarius* survival. The most visible changes will be the shift in range to the north in the eastern part of the European plain. The comfort conditions for *P. melanarius* decrease in mountain regions, including the Alps, Carpathians, Caucasus, and Urals. By 2070, the coenotic optimum will significantly decrease on the Balkan Peninsula. Thus, a sharp reduction in Southern European and Mediterranean populations has been predicted [51].

### 2.2. Sampling Area

**Figure 1:**
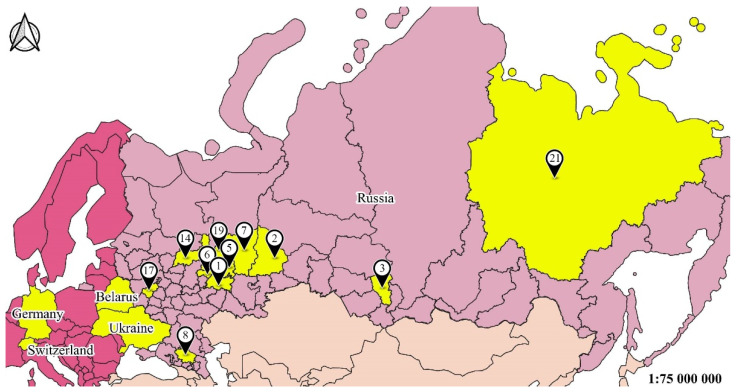
Map of the study. Note: Sampling localities; see Table 1.

The sampling area included vast territory in different provinces of Russia, including Siberia, and several sites in four European countries (Table 1, Figure 1).

As seen from Table 1, the studied localities differed by 17 degrees in latitude and 121 degrees in longitude. In relation to latitude, we considered the difference sufficient according to the several papers devoted to latitude gradient investigations in different invertebrate taxa life traits [52,53,54]. We sampled beetles in at least three plots in every studied region. Those plots differed in some parameters: anthropogenic impact, vegetation, etc. However, we took into account that our investigation was large in scale. Due to the huge number of sampled and measured beetles, we performed the study by developing a high-throughput approach. This kind of approach has become popular in recent decades. It involves gathering data from scattered publications on the definite item, colligating it, and making conclusions on the given topic, e.g., [29].

### 2.3. Study Design

Beetles were sampled in the given regions in 2010–2018. All beetles captured in each locality during that period formed the dataset for morphometric analysis for that locality. Studied animals were captured using pitfall traps with saline solution. Each sampling plot comprised a direct line of traps with over 10 m distance between each. Sampling trap exposition lasted approximately 5 days. Subsequently, animals were preserved in 70% alcohol prior to taxonomic differentiation and were deposited straightened onto cotton padding thereafter.

The number of insects collected in each region was equal to the number of insects analyzed morphologically. Thus, the numbers in the last column of Table 1 are the numbers of measured beetles.

**Figure 2:**
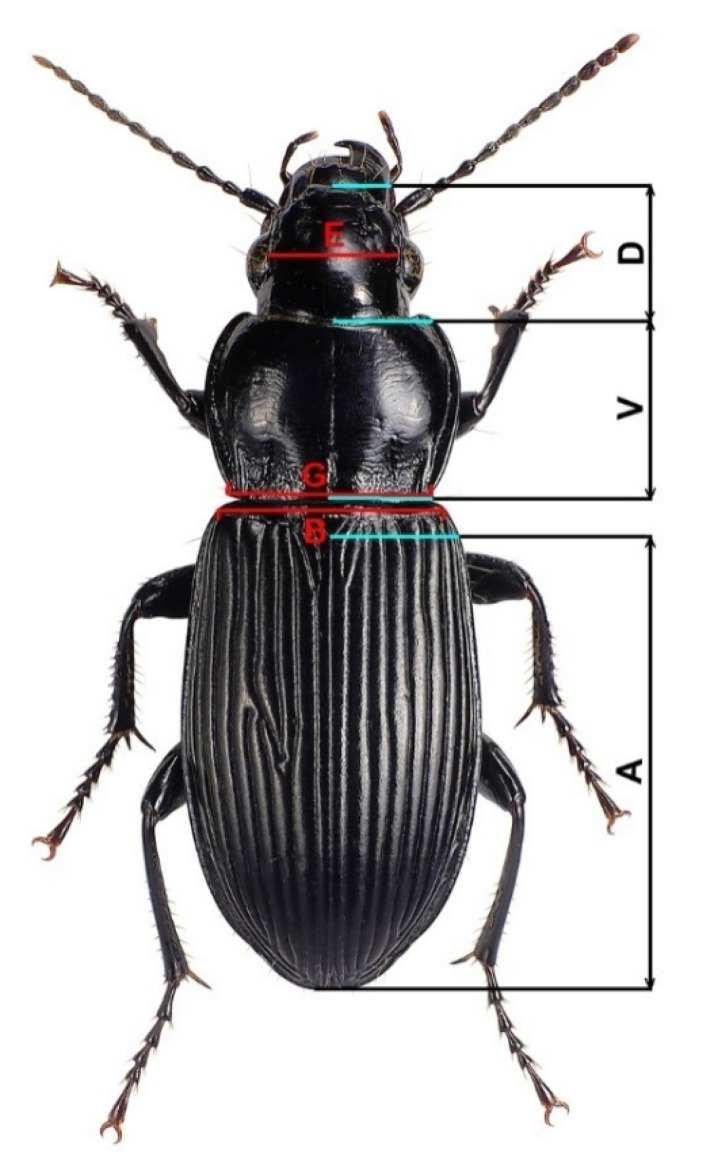
Placement of morphometric measurements. A—Elytra length as distance between posterior end of scutellum and terminus of right elytron (in the case of absence of intact right elytron, left one is acceptable). B—Elytra width as distance between anterior-distal corners of elytra. V—Pronotum length measured along of central furrow. G—Pronotum width as distance between posterior corners of the pronotum. D—Head length as distance between labrum and juncture of occiput and postgena. E—Head width as distance between proximal innermost sides of eyes. Measured sample size is presented in Table 1.

All paddings with captured beetles were transferred to the Laboratory of Biomonitoring (The Institute of Problems in Ecology and Mineral Wealth, Tatarstan Academy of Sciences) for photographing. All photos were taken by one person using the same method. Morphometric data were collected from images taken by a Nikon D5100 camera with a custom opaque light disperser and a box with an opaque reflective surface. Measurements were taken using a program designated specifically for the given method of measurement and utilized a distance between manually pointed out elements of photo arrays as the terminal point of measurements and the fiducial scale, using the latter to bind the real scale to the array output data. Thus, the measuring was done using “Manual Carabid morphometric measurement for the method by Sukhodolskaya”. Initial codes from the latter are available under the free permissive license MIT [55]. The utilized morphometric data corresponded to six linear scalar float recordings as dependent variables and were given for following values (denoting letters are given as Cyrillic transliteration of reference material’s source) (Figure 2).

### 2.4. Statistical Analysis

A linear regression model was used to assess the relationship between ground beetle size and geographic coordinates:

$$Y_{k,i}=a_{k,0}+a_{k,1}\cdot I_{male}\left(Sex_{i}\right)+a_{k,2}\cdot Coord_{i}+a_{k,3}\cdot I_{male}\left(Sex_{i}\right)\cdot Coord_{i}+{Ɛ}_{k,i}$$

where *k* is the index of the dimension (A—Elytra Length, B—Elytra Width, V—Pronotum Length, G—Pronotum Width, D—Head Length, E—Eye Distance), *i* is the index of the ground beetle specimen, *Y_k_*,_*i*_ is the dimension value, *I_male_*(*Sex_i_*) is the indicator function that takes a value of 0 for females and 1 for males, *Coord_i_* is the geographic coordinate (longitude or latitude), Ɛ*_k_*,_*i*_ is the random error, and *a_k_*_,0_, *a_k_*_,1_, *a_k_*_,2_, *a_k_*_,3_ are the coefficients of the model.

The *a_k_*_,0_ coefficient is the model intercept for females, the *a_k_*_,1_ coefficient is a correction to it for males, *a_k_*_,2_ is the slope of the regression for females, and *a_k_*_,3_ is an amendment to it for males, which allows us to estimate the sexual dimorphism in the dependence of size on geographic coordinates and its significance.

## 3. Results

**Figure 3:**
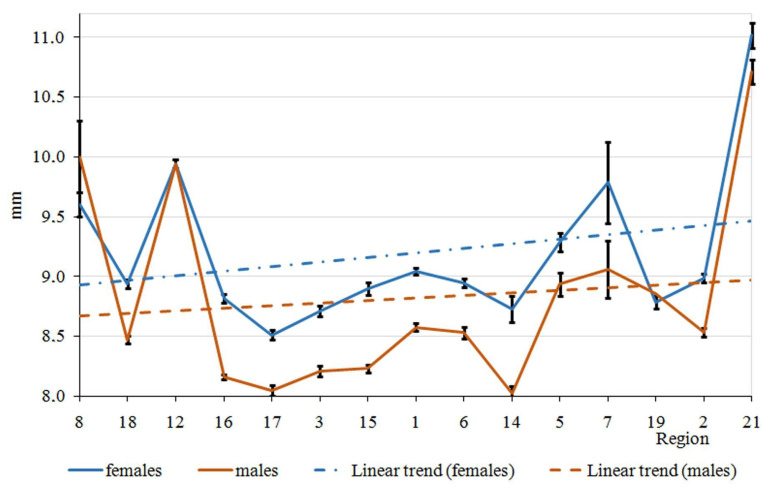
Elytra length variation in latitude gradient in *P. melanarius*.

**Figure 4:**
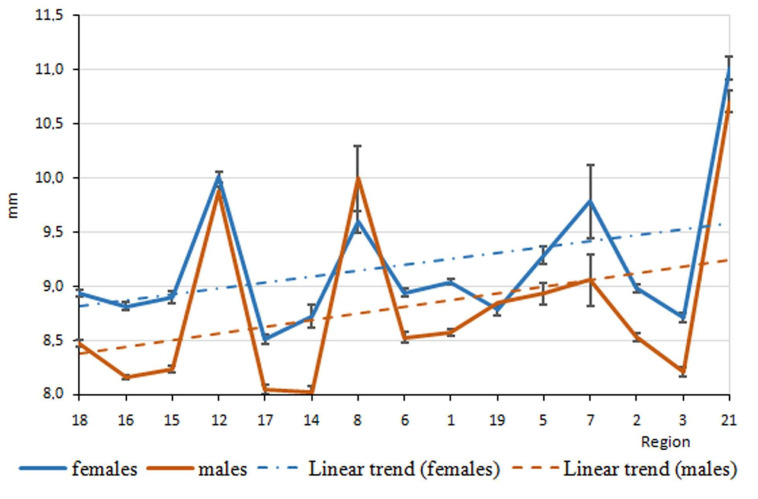
Elytra length variation in longitude gradient in *P. melanarius*.

**Figure 5:**
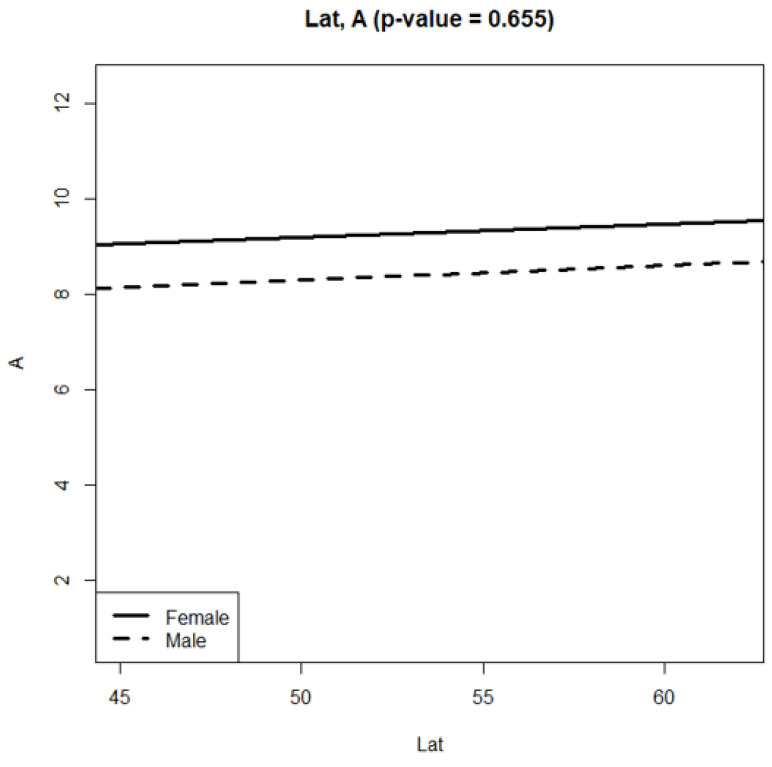
Regression slopes of elytra length variation in latitude gradient in *P. melanarius*.

**Figure 6:**
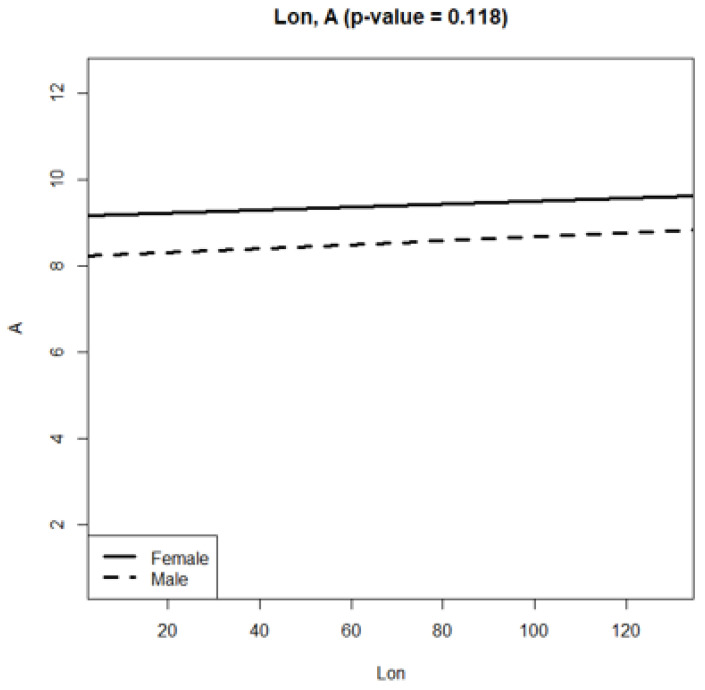
Regression slopes of elytra length variation in longitude gradient in *P. melanarius*.

The elytra length variation in *P. melanarius* by latitude gradient was sawtooth (Figure 3); trait values sharply changed between the 8th and 16th regions, situated within 5° in latitude. A relatively smooth curve between 51° and 57° (the regions designated as 16, 17, 3, 15, 1, 6, 14 on the horizontal axes) gave way to a sharp rise in the trait values in the populations at the regions practically at the same latitude (regions 5, 7). Then, elytra length decreased again and sharply increased in the most northern region (21). The longitude gradient in elytra length variation was no less dramatic (Figure 4). However, the trends of trait increase were statistically significant in both gradients (Table 2 and Table 3). In addition, they did not differ in males and females (Figure 5 and Figure 6). It was common for both gradients that males were smaller than females (excluding populations of *P. melanarius* in 3 studied regions out of 15).

Sawtooth patterns were more strongly expressed in the other trait variations (Figures S1–S10): the values changed in the populations of neighboring regions in latitude gradient and the longitude as well. Regression coefficients were significant in all cases, excluding pronotum length variation in both gradients and elytra width variation in longitude (Tables S2, S6, S7). Thus, in the latitude gradient, elytra parameters increased (Figure 2 and Figure S1, Table 2 and Table S1), and pronotum length did not change but the width increased (Tables S2 and S3). Head parameters decreased (Tables S4 and S5). In the longitude gradient, the changes were as follows: elytra length increased (Table 2), but its width did not change (Table S6); pronotum length did not change, but its width increased (Tables S7 and S8); the head parameters decreased (Tables S9 and S10). Thus, we observed an elytra length increase and head parameter decrease northwards and eastwards.

We mention that the trends of the latitude and longitude elytra length variation were similar in both sexes because p-levels were not significant when comparing female and male regression curves. We compared these curves in relation to the other traits studied (Figures S11–S20): p-levels were significant only in four cases—in relation to pronotum parameter variation in the latitude gradient (Figures S12 and S13) and elytra width and the pronotum length variation in the longitude gradient (Figures S16 and S17). Therefore, in general, there was no evidence for the greatest steepness in trait variation in males compared with females.

## 4. Discussion

We did not find regular body size variation for *P. melanarius* by latitude or longitude gradients in relation to all six treated traits. Only elytra and pronotum length curves were U-shaped. Similar results were obtained in a study of damsfly body size variation, where insects were larger at the northern and southern edges of the area [56]. The authors explained that fact by the facilitating dispersal ability and increasing investment in reproduction via fecundity. However, they used the single parameter as the proxy of body size. In our study, elytra width increased by latitude gradient and decreased by longitude. The other traits varied in a sawtooth manner. In this case, our results are in accordance with those of researchers who investigated wasps intraspecific body size (the range in latitude was 20° and in longitude 30°). It was correlated with latitude, elevation, and broad-scale climate variation. However, the direction of that relationship was idiosyncratic across species, with Bergmann’s clines and converse Bergmann’s clines equally represented. There was no evidence of phylogenetic signal in the direction of the cline between body size and the environment [57].

We consider that in our case the main explanatory variable for body size variation was the study region. In this relation, we agree with Nils Hein′s studies of *P. palustris*: its body size variation showed a somehow curvilinear pattern, probably related to the transition zones between the elevational belts. In both sexes, the explanatory variables, sorted by their importance in decreasing order, were (1) study region; (2) elevation; (3) topographic position [58]. It is important to note that topographical position remained in the last position. In this view, our hypotheses is in agreement: in large-scale studies, the impact of local highly diverse factors should not be taken into account due to the fact that it would distort the general pattern. Widespread species are exposed to a variety of environmental conditions throughout their distribution range, often resulting in local adaptations [59]. Spatial variation in fitness-related traits often reflects differences in selective pressures, which determine these adaptations [60,61,62]. Clinal variation may indicate such local adaptation, as geographical clines are strongly related to environmental gradients, which may pose differential challenges to survival and reproduction. In addition, animals may also exhibit striking body size variation at smaller spatial scale for nearby locations with similar climate characteristics but with variation in microhabitat conditions, such as the nature of the substrate, which may impose nutritional and/or hydric constraints [63].

It is worth noting the differences in males and females in geographical body size variation. In a review based on results of a large number of publications, it was found that males’ latitudinal body size clines were steeper than those of females [29]. This resulted in greater among-population variation in male than female body size, and the magnitude of the allometric slope between male and female size increased significantly with the ratio of male to female latitudinal clines in body size (but not with dimorphism). The authors focused on the relationship between dimorphism allometry and sex-specific latitudinal clines. However, a weak part of the work was that the authors used information that was not obtained independently. For many cases, it might be that the establishment of consistent genetic differences in dimorphism among populations, presumably due to sex-specific selection [64,65], is likely hampered by factors such as phenotypic plasticity [66], gene flow between populations [67], temporal variability in selection on male or female size, e.g., [68], or nonequilibrium dimorphism arising from the generally very high genetic correlation between male and female size [69]. Clines also vary over time, which is especially relevant for quickly evolving organisms such as insects. In addition, carabids’ body size depends on various local factors (nutrient source, wintering conditions, soil humidity, vegetation, etc.). This is why the data set of our research included samples from several seasons in certain latitudes, different types of biotopes, and the anthropogenic impact in each province. We argue that the types of beetles’ body part variation are the result of latitude impact. There are significant issues when researchers gather insect body size data not from specimen measuring but from previous literature, where body size was an ancillary data point in unrelated experiments (see Blanckenhorn et al. [29]). In our study, all measurements were performed by a single researcher staff according to a single method. We did not find that male variation in body size was steeper that female variation.

Second, another approach should be used additionally to solve the problem of similarity in body size variation in different sexes. Expanding the range of our research, we intend to follow some researchers who used neighboring latitude regions [30]. Accordingly, traits shifts and their significance were estimated for two neighboring regions. In addition, they could not apply the simple regression analysis in their case for the reasons that body size variation in the species studied could be sawtooth, as was shown for the ground beetle in our earlier study on *Pterostichus melanarius* [16].

Nevertheless, identifying the putative microevolutionary mechanisms causing geographical clines among species requires studying variation in dimorphism among populations in detail and in a greater number of species. In our case, we do not solely focus on latitude/longitude clines. Localities differ in climatic and local factors, affecting the body size of the insects. The aim of the study was to trace the steepness of trait variation between the localities studied, and the steepness was found to be equal in males and females.

## 5. Conclusions

In the face of natural and anthropogenic climate change, investigating widespread species to show local variation is important, as such species may respond differently across their distribution range to ongoing environmental changes, depending on their genetically based plasticity capacities. Overall, our study identified an interesting pattern in life-history strategies, with sawtooth body size variation, which was expressed equally in males and females.

## Figures and Tables

**Table 1:** Sampling localities and sample size.

N	Region	Latitude, °N	Longitude, °E	Number of Sites	Type of Habitats	Sample Size
1 ^1^	Tatarstan Republic	55°47′	49°06′	53	Meadow, birch, oak, elm	11,312
2	Sverdlovsk region	58°42′	61°20′	6	Meadow, pine, birch	458
3	Kemerovo region	54°56′	87°14′	20	Meadows, birch, lawn	1954
5	Udmurtia Republic	57°17′	52°45′	16	Birch, oak, elm	396
6	Mariy El Republic	56°42′	47°52′	14	Meadow, birch, oak	67
7	Cis-Ural	57°01′	57°9′	21	Birch, oak, elm	58
8	Stavropol region	45°02′	41°55′	6	Meadow, birch	76
12	Ukraine	48°27′	34°56′		Artificial forest plantation	225
14	Kostroma region	57°48′	41°19′	3	Fir dominated ecosystem recovery after felling	60
15	Belarus	55°13′	30°18′	3	Forest with pine, oak, alder, willow	369
16	Germany	52°45′	9°23′	18	Oilseed rape fields	1339
17	Kaluga region	54°32′	36°16′	8	Gardens, old-growth forest	233
18	Switzerland	47°23′	8°05′	8	Oilseed rape fields	450
19	Kirov region	58°001′	48°027′	3	Spruce, lime and oak forests	57
21	Yakutsk region	62°01′	129°43′	2	Meadows, lawn	48

^1^ In the first column are the numbers that corresponded to the number in the database of our laboratory. The latter includes newly studied regions. In some regions, we did not record *P. melanarius*, so these regions were not included in the analysis. Therefore, the numbers in the first column are not continuous. These numbers are included in Figure 1 and are noted on the X-axis.

**Table 2:** Regression results in latitude elytra length variation in *P. melanarius*.

	Estimate	Std. Error	t Value	Pr(>|t|)	
(Intercept)	7.8008	0.1933	40.350	<2 × 10^−16^	***
fSexMale	−0.9916	0.2692	−3.683	0.0002	***
Lat	0.0276	0.0035	7.869	4.16 × 10^−15^	***
fSexMale:Lat	0.0022	0.0049	0.447	0.6549	

Notes. fSexMale—how males differed in trait size from females (here we see that they were smaller); Lat—significance of regression coefficient in females (latitude affected trait size in positive direction); fSexMale:Lat—difference in regression coefficients between males and females (it was insignificant); *** *p*-level < 0.001.

**Table 3:** Regression results in longitude elytra length variation in *P. melanarius*.

	Estimate	Std. Error	t value	Pr(>|t|)	
(Intercept)	9.1650	0.0301	303.632	<2 × 10^−16^	***
fSexMale	−0.9406	0.0418	−22.488	<2 × 10^−16^	***
Lon	0.0032	0.0005	5.561	2.8 × 10^−8^	***
fSexMale:Lon	0.0012	0.0008	1.564	0.118	

Note: see Table 2. *** *p*-level < 0.001.

## Data Availability

Not applicable.

## Supplementary Materials

The following supporting information can be downloaded at: https://www.mdpi.com/article/10.3390/life12010112/s1, Figure S1: Elytra width variation in latitude gradient in *P. melanarius*. Figure S2: Pronotum length variation in latitude gradient in *P. melanarius*. Figure S3: Pronotum width variation in latitude gradient in *P. melanarius.* Figure S4: Head length variation in latitude gradient in *P. melanarius*. Figure S5: Distance between eyes variation in latitude gradient in *P. melanarius*. Figure S6: Elytra width variation in longitude gradient in *P. melanarius*. Figure S7: Pronotum length variation in longitude gradient in *P. melanarius*. Figure S8: Pronotum width variation in longitude gradient in *P. melanarius*. Figure S9: Head length variation in longitude gradient in *P. melanarius*. Figure S10: Distance between eyes variation in longitude gradient in *P. melanarius*. Figure S11: Regression slopes of elytra width variation in latitude gradient in *P. melanarius*. Figure S12: Regression slopes of pronotum length variation in latitude gradient in *P. melanarius*. Figure S13: Regression slopes of pronotum width variation in latitude gradient in *P. melanarius*. Figure S14: Regression slopes of head length variation in latitude gradient in *P. melanarius*. Figure S15: Regression slopes of distance between eyes variation in latitude gradient in *P. melanarius*. Figure S16: Regression slopes of elytra width variation in longitude gradient in *P. melanarius*. Figure S17: Regression slopes of pronotum length variation in longitude gradient in *P. melanarius*. Figure S18: Regression slopes of pronotum width variation in longitude gradient in *P. melanarius*. Figure S19: Regression slopes of head length variation in longitude gradient in *P. melanarius*. Figure S20: Regression slopes of distance between eyes variation in longitude gradient in *P. melanarius*. Table S1: Regression results in latitude elytra width variation in *P. melanarius*. Table S2: Regression results in latitude pronotum length variation in *P. melanarius*. Table S3: Regression results in latitude pronotum width variation in *P. melanarius*. Table S4: Regression results in latitude head length variation in *P. melanarius*. Table S5: Regression results in latitude distance between eyes variation in *P. melanarius*. Table S6: Regression results in longitude elytra width variation in *P. melanarius*. Table S7: Regression results in longitude pronotum length variation in *P. melanarius*. Table S8: Regression results in longitude pronotum width variation in *P. melanarius*. Table S9: Regression results in longitude head length variation in *P. melanarius*. Table S10: Regression results in longitude distance between eyes variation in *P. melanarius*.
