# Supplementary material for: Modeling Sexual Differences of Body Size Variation in Ground Beetles in Geographical Gradients: A Case Study of Pterostichus melanarius (Illiger, 1798) (Coleoptera, Carabidae)
Source: Life (Basel). 2022 Jan 13;12(1):112. doi: 10.3390/life12010112 (PMC8781924; doi:10.3390/life12010112)
Supplement: Supplementary file 1 [file life-12-00112-s001.zip › list of figures.pdf]

Figures S

S1 – Elytra width variation in latitude gradient in *P. melanarius*
S2 – Pronotum length variation in latitude gradient in *P. melanarius*
S3 – Pronotum width variation in latitude gradient in *P. melanarius*
S4 – Head length variation in latitude gradient in *P. melanarius*
S5 – Distance between eyes variation in latitude gradient in *P. melanarius*
S6 – Elytra width variation in longitude gradient in *P. melanarius*
S7 – Pronotum length variation in longitude gradient in *P. melanarius*
S8 – Pronotum width variation in longitude gradient in *P. melanarius*
S9 – Head length variation in longitude gradient in *P. melanarius*
S10 – Distance between eyes variation in longitude gradient in *P. melanarius*
S11 – Regression slopes of elytra width variation in latitude gradient in *P. melanarius*
S12 – Regression slopes of pronotum length variation in latitude gradient in *P. melanarius*
S13 – Regression slopes of pronotum width variation in latitude gradient in *P. melanarius*
S14 – Regression slopes of head length variation in latitude gradient in *P. melanarius*
S15 – Regression slopes of distance between eyes variation in latitude gradient in *P. melanarius*
S16 – Regression slopes of elytra width variation in longitude gradient in *P. melanarius*
S17 – Regression slopes of pronotum length variation in longitude gradient in *P. melanarius*
S18 – Regression slopes of pronotum width variation in longitude gradient in *P. melanarius*
S19 – Regression slopes of head length variation in longitude gradient in *P. melanarius*
S20 – Regression slopes of distance between eyes variation in longitude gradient in *P. melanarius*
