# Supplementary material for: Modeling Sexual Differences of Body Size Variation in Ground Beetles in Geographical Gradients: A Case Study of Pterostichus melanarius (Illiger, 1798) (Coleoptera, Carabidae)
Source: Life (Basel). 2022 Jan 13;12(1):112. doi: 10.3390/life12010112 (PMC8781924; doi:10.3390/life12010112)
Supplement: Supplementary file 1 [file life-12-00112-s001.zip › Tables_Supplementary materials.pdf]

life MDPI

Table S1. Regression results in latitude elytra width variation in *P. melanarius*.

	Estimate	Std. Error	t value	Pr(>ǀtǀ)	
(Intercept)	5.2518	0.1990	26.379	< 2e-16	***
fSexMale	-0.0757	0.2703	-0.280	0.779	
Lat	-0.0339	0.0036	-9.400	< 2e-16	***
fSexMale:Lat	0.0029	0.0049	0.606	0.544	

Notes. fSexMale – how males differed in trait size from females (here we see that they were smaller); Lat – significance of regression coefficient in females (latitude really affected trait size in positive direction); fSexMale:Lat – difference in regression coefficients between males and females (it was insignificant); ***– p-level<0.001.

Table S2. Regression results in latitude pronotum length variation in *P. melanarius*.

	Estimate	Std. Error	t value	Pr(>ǀtǀ)	
(Intercept)	3.3079	0.086	38.430	< 2e-16	***
fSexMale	-0.8671	0.1198	-7.233	5.27e-13	***
Lat	0.0004	0.0015	0.316	0.752	
fSexMale:Lat	0.0123	0.0021	5.622	1.97e-08	***

Notes. The same as in the Table S1.

Table S3. Regression results in latitude pronotum width variation in *P. melanarius*.

	Estimate	Std. Error	t value	Pr(>ǀtǀ)	
(Intercept)	3.1597	0.1071	29.481	< 2e-16	***
fSexMale	-0.6717	0.1492	-4.500	6.93e-06	***
Lat	0.0069	0.0019	3.556	0.0003	***
fSexMale:Lat	0.0075	0.0027	2.742	0.0061	**

Notes. The same as in the Table S1; **– p-level<0.01.

Table S4. Regression results in latitude head length variation in *P. melanarius*.

	Estimate	Std. Error	t value	Pr(>ǀtǀ)	
(Intercept)	7.8025	0.1351	57.728	< 2e-16	***
fSexMale	-0.3897	0.1882	-2.070	0.0385	*
Lat	-0.0953	0.0024	-38.744	<2e-16	***
fSexMale:Lat	0.0043	0.0034	1.249	0.2119	

Notes. The same as in the Table S1; *– p-level<0.05.

Table S5. Regression results in latitude distance between eyes variation in *P. melanarius*.

	Estimate	Std. Error	t value	Pr(>ǀtǀ)	
(Intercept)	2.5991	0.0751	34.568	< 2e-16	***
fSexMale	-0.1283	0.102	-1.257	0.209	
Lat	-0.0057	0.0013	-4.191	2.82e-05	***
fSexMale:Lat	-0.001	0.0018	-0.587	0.558	

Notes. The same as in the Table S1.

Table S6. Regression results in longitude elytra width variation in *P. melanarius*.

	Estimate	Std. Error	t value	Pr(>|t|)	
(Intercept)	3.380e+00	3.068e-02	110.168	< 2e-16	***
fSexMale	2.094e-01	4.188e-02	5.000	5.88e-07	***
Lon	7.459e-05	5.903e-04	0.126	0.8995	
fSexMale:Lon	-1.983e-03	8.191e-04	-2.421	0.0155	*

Notes. The same as in the Table S1; *– p-level<0.05.

Table S7. Regression results in longitude pronotum length variation in *P. melanarius*.

	Estimate	Std. Error	t value	Pr(>|t|)	
(Intercept)	3.3418	0.0134	248.514	< 2e-16	***
fSexMale	-0.2561	0.0186	-13.744	< 2e-16	***
Lon	-0.0001	0.0002	-0.546	0.5847	
fSexMale:Lon	0.0012	0.0003	3.445	0.0005	***

Notes. The same as in the Table S1.

Table S8. Regression results in longitude pronotum width variation in *P. melanarius*.

	Estimate	Std. Error	t value	Pr(>|t|)	
(Intercept)	3.4601	0.0166	207.621	< 2e-16	***
fSexMale	-0.2942	0.023	-12.739	< 2e-16	***
Lon	0.0017	0.0003	5.221	1.84e-07	***
fSexMale:Lon	0.0006	0.0004	1.401	0.161	

Notes. The same as in the Table S1.

Table S9. Regression results in longitude head length variation in *P. melanarius*.

	Estimate	Std. Error	t value	Pr(>|t|)	
(Intercept)	3.0522	0.0235	129.812	< 2e-16	***
fSexMale	-0.1	0.0325	-3.071	0.0021	**
Lon	-0.0101	0.0004	-22.046	< 2e-16	***
fSexMale:Lon	-0.0002	0.0006	-0.418	0.6756	

Notes. The same as in the Table S1; **– p-level<0.01.

Table S10. Regression results in longitude distance between eyes variation in *P. melanarius*.

	Estimate	Std. Error	t value	Pr(>|t|)	
(Intercept)	2.330e+00	1.145e-02	203.511	< 2e-16	***
fSexMale	-1.837e-01	1.563e-02	-11.755	< 2e-16	***
Lon	-9.462e-04	2.203e-04	-4.295	1.77e-05	***
fSexMale:Lon	-3.836e-05	3.056e-04	-0.126	0.9	

Notes. The same as in the Table S1.
