# Supplementary figures and images for: Modeling Sexual Differences of Body Size Variation in Ground Beetles in Geographical Gradients: A Case Study of Pterostichus melanarius (Illiger, 1798) (Coleoptera, Carabidae)
Source: Life (Basel). 2022 Jan 13;12(1):112. doi: 10.3390/life12010112 (PMC8781924; doi:10.3390/life12010112)

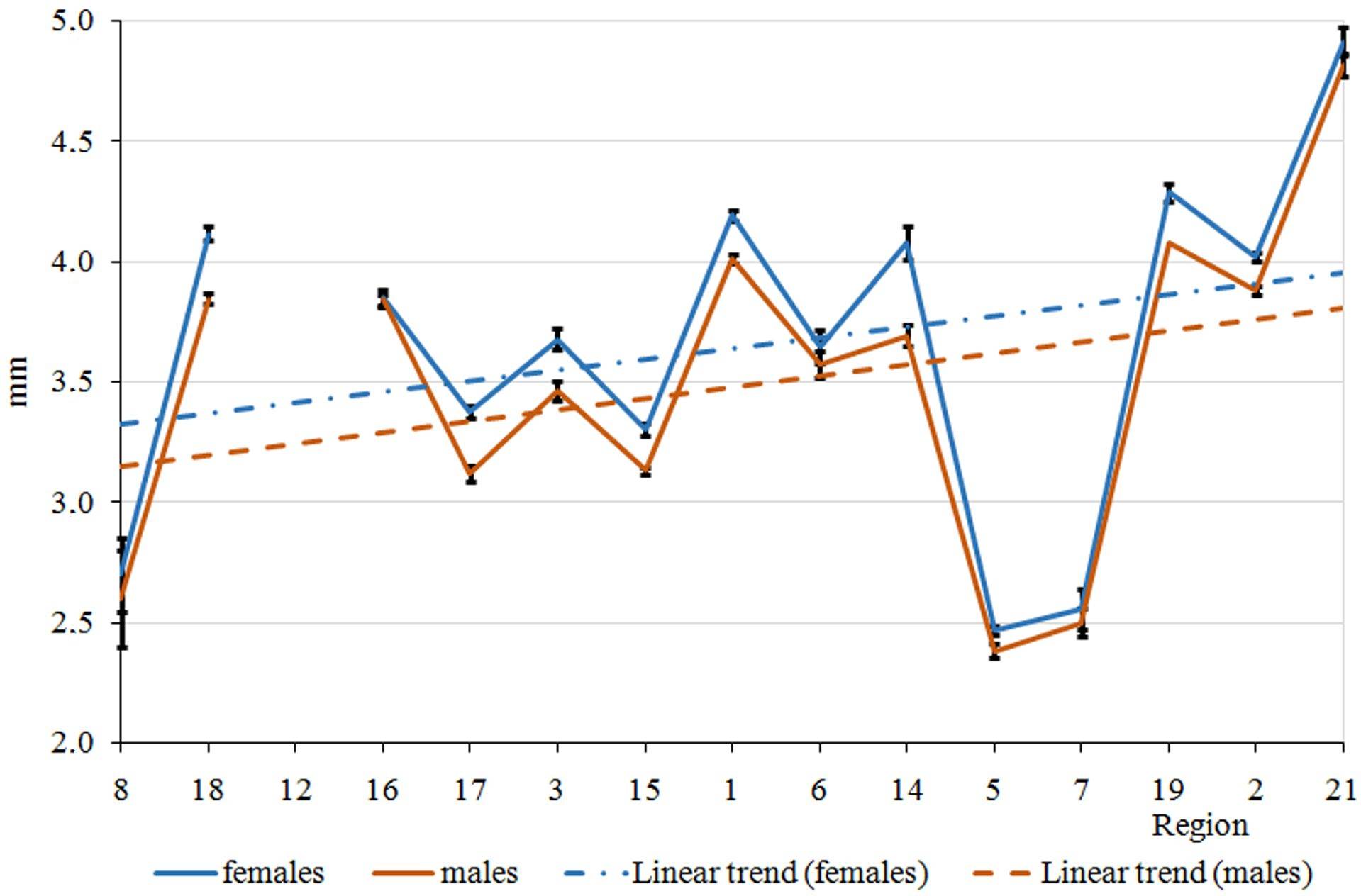

Supplement: Supplementary file 1 [file life-12-00112-s001.zip › S 1.jpg]

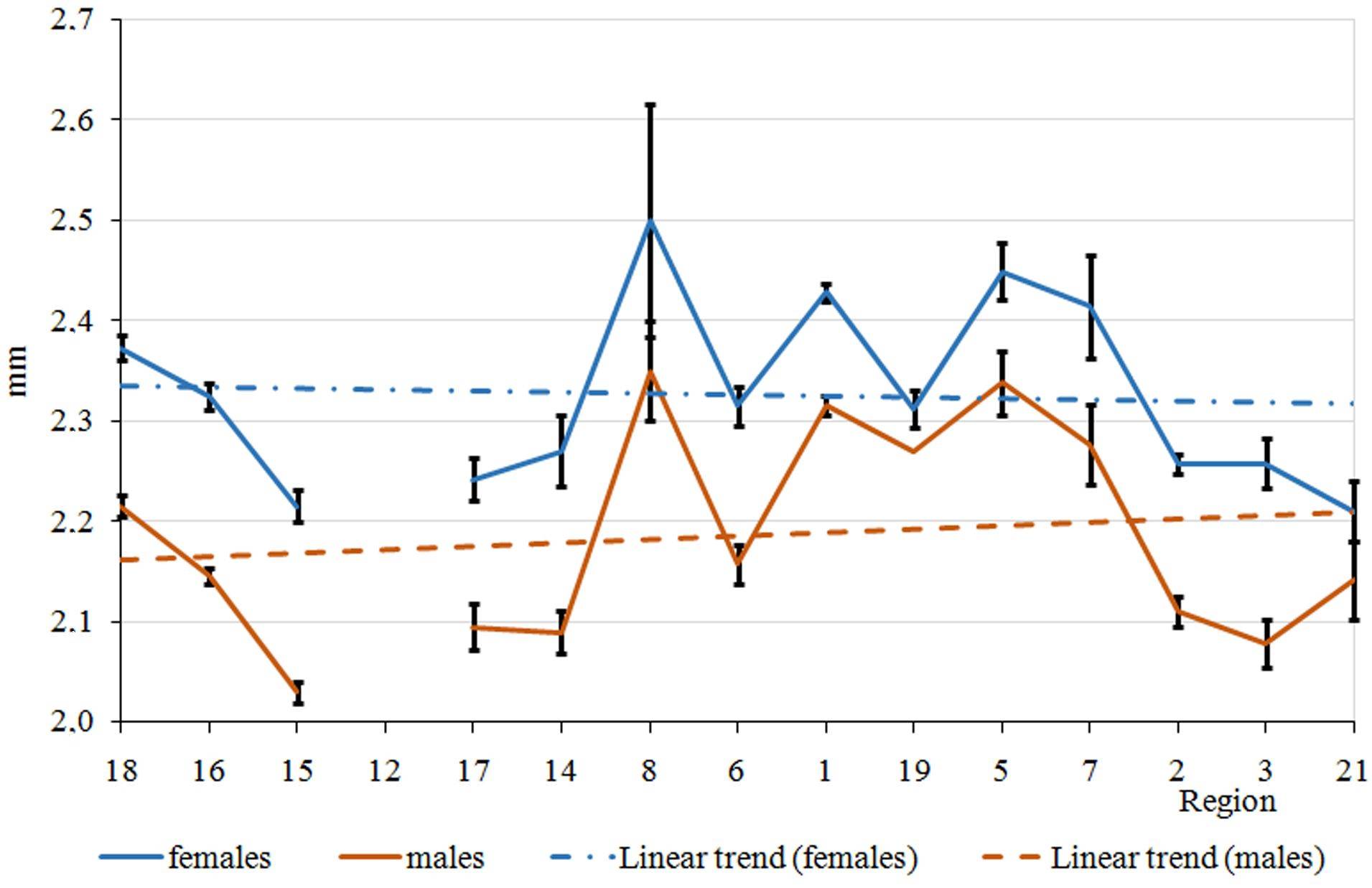

Supplement: Supplementary file 1 [file life-12-00112-s001.zip › S 10.jpg]

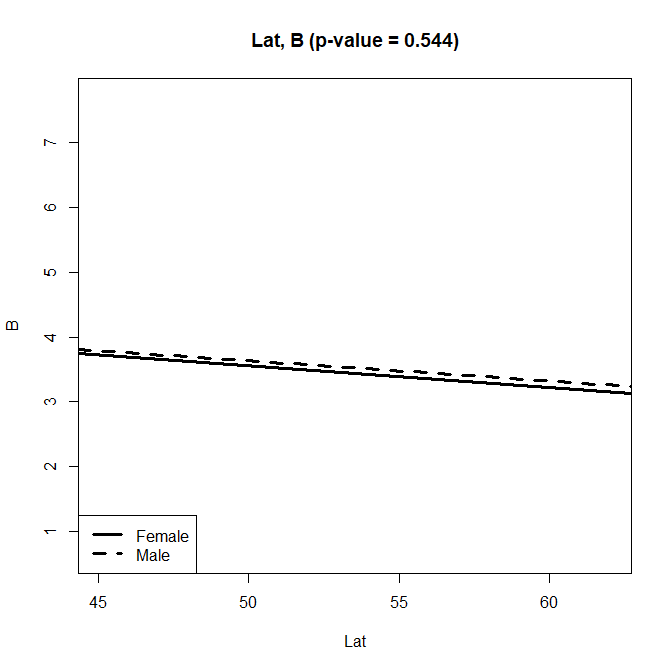

Supplement: Supplementary file 1 [file life-12-00112-s001.zip › S 11.png]

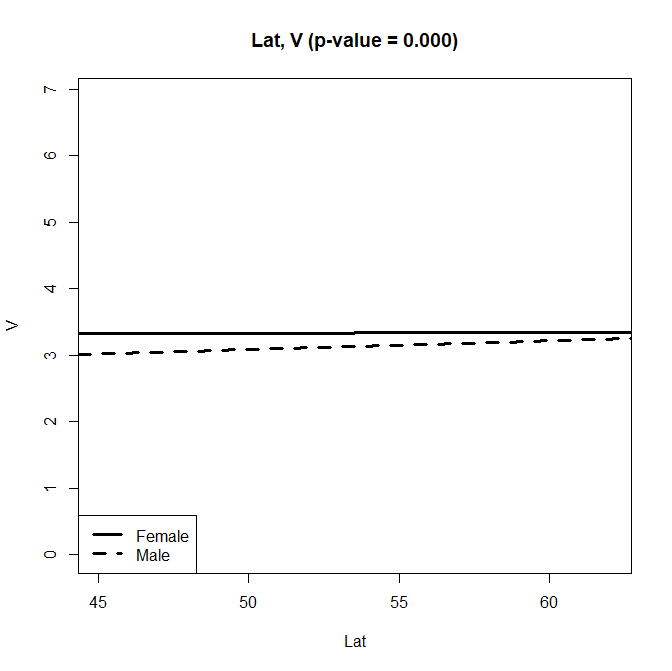

Supplement: Supplementary file 1 [file life-12-00112-s001.zip › S 12.png]

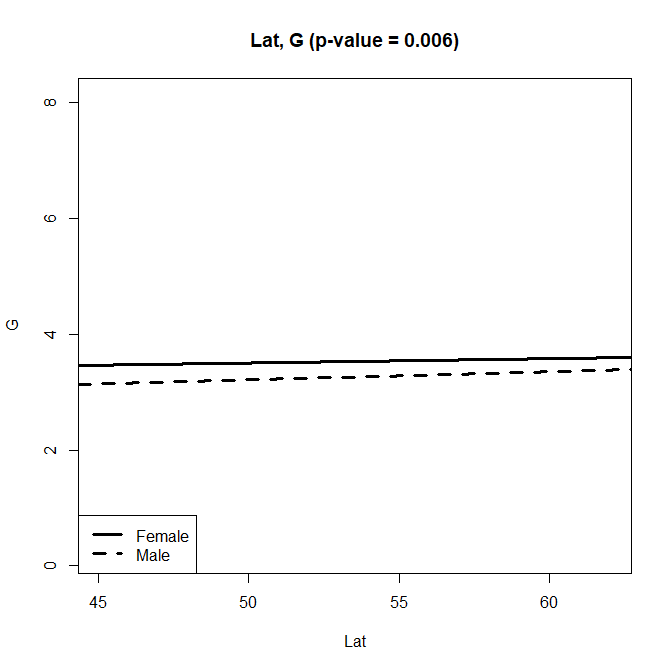

Supplement: Supplementary file 1 [file life-12-00112-s001.zip › S 13.png]

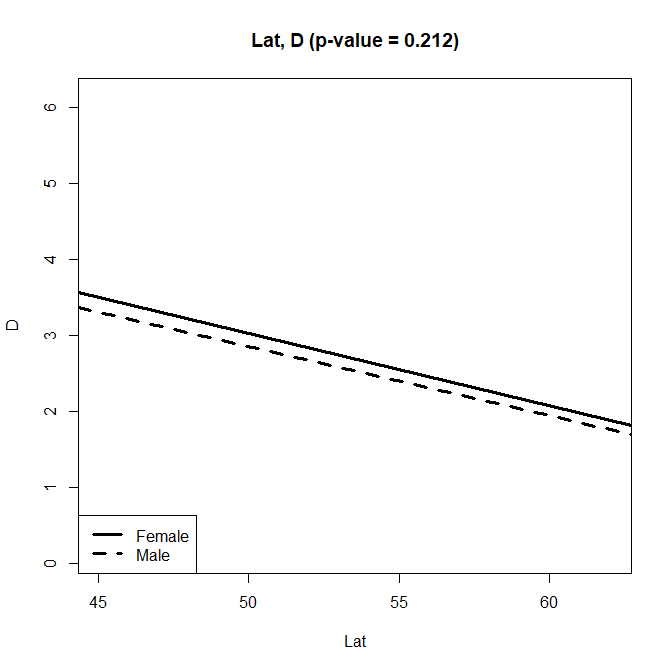

Supplement: Supplementary file 1 [file life-12-00112-s001.zip › S 14.png]

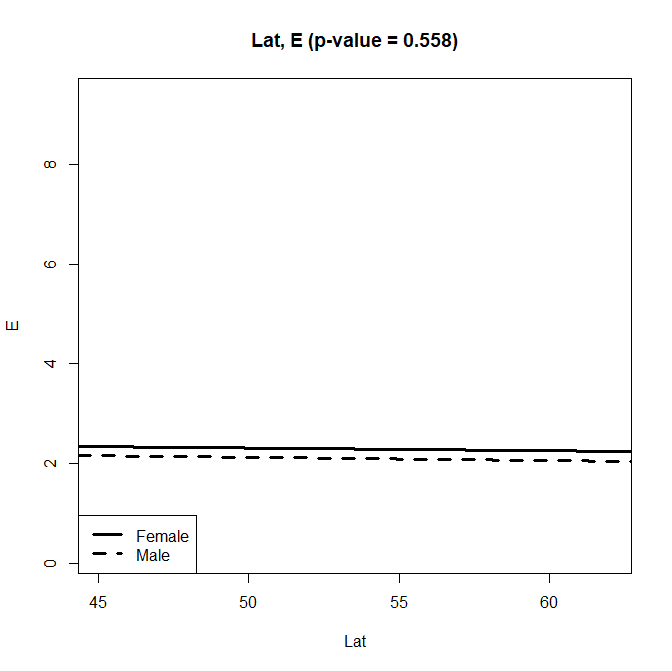

Supplement: Supplementary file 1 [file life-12-00112-s001.zip › S 15.png]

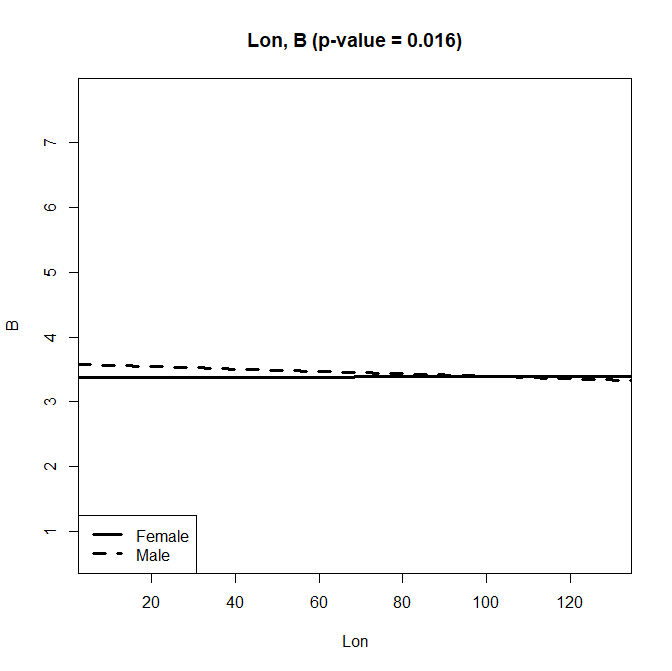

Supplement: Supplementary file 1 [file life-12-00112-s001.zip › S 16.png]

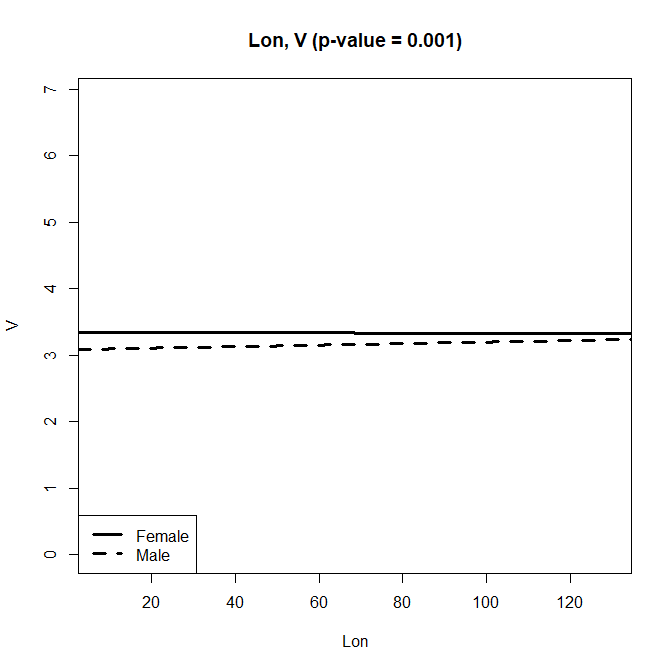

Supplement: Supplementary file 1 [file life-12-00112-s001.zip › S 17.png]

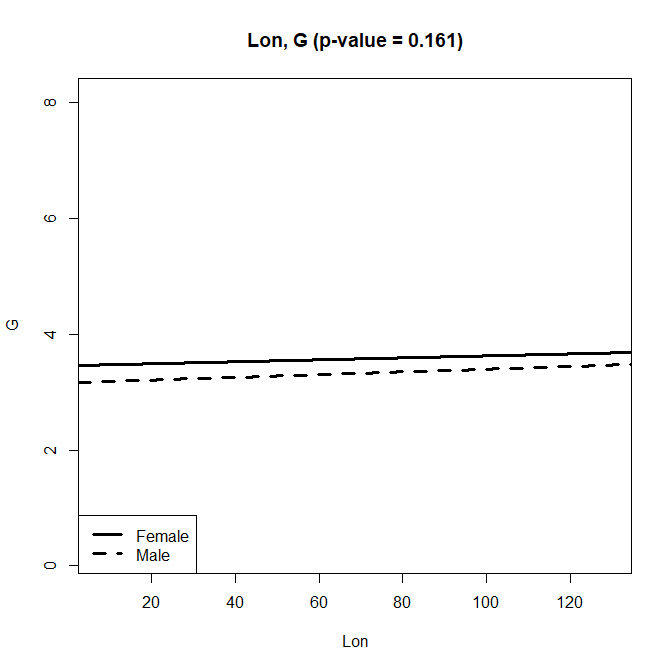

Supplement: Supplementary file 1 [file life-12-00112-s001.zip › S 18.png]

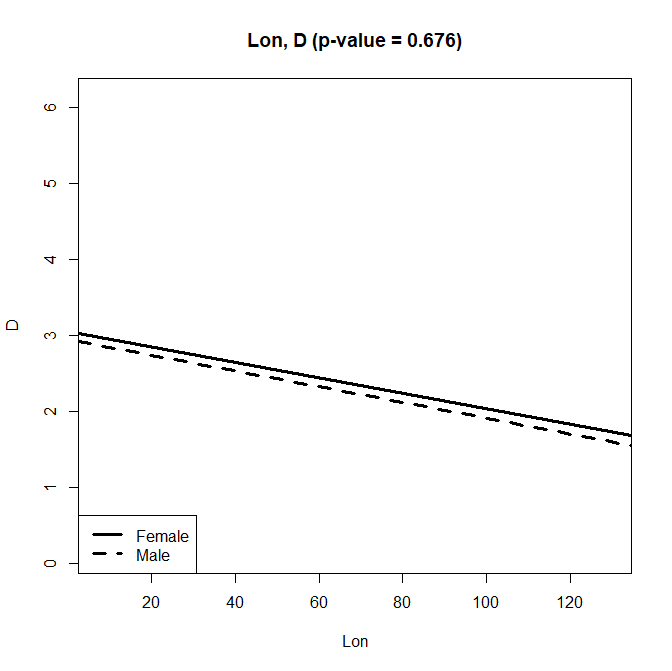

Supplement: Supplementary file 1 [file life-12-00112-s001.zip › S 19.png]

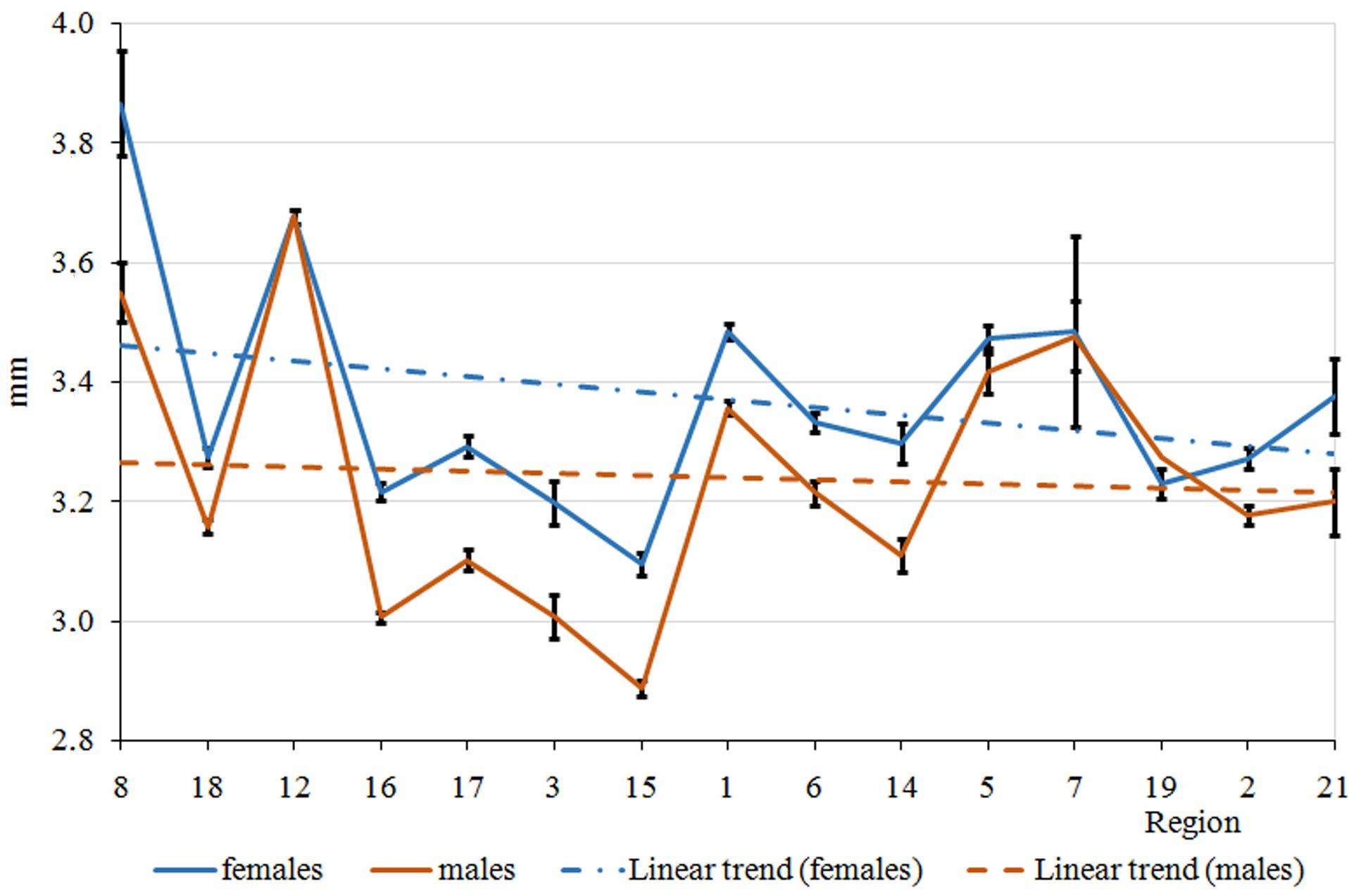

Supplement: Supplementary file 1 [file life-12-00112-s001.zip › S 2.jpg]

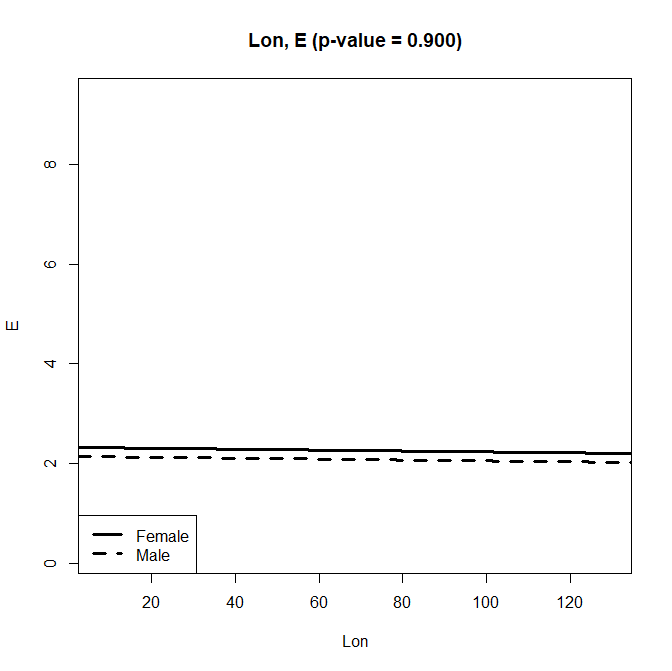

Supplement: Supplementary file 1 [file life-12-00112-s001.zip › S 20.png]

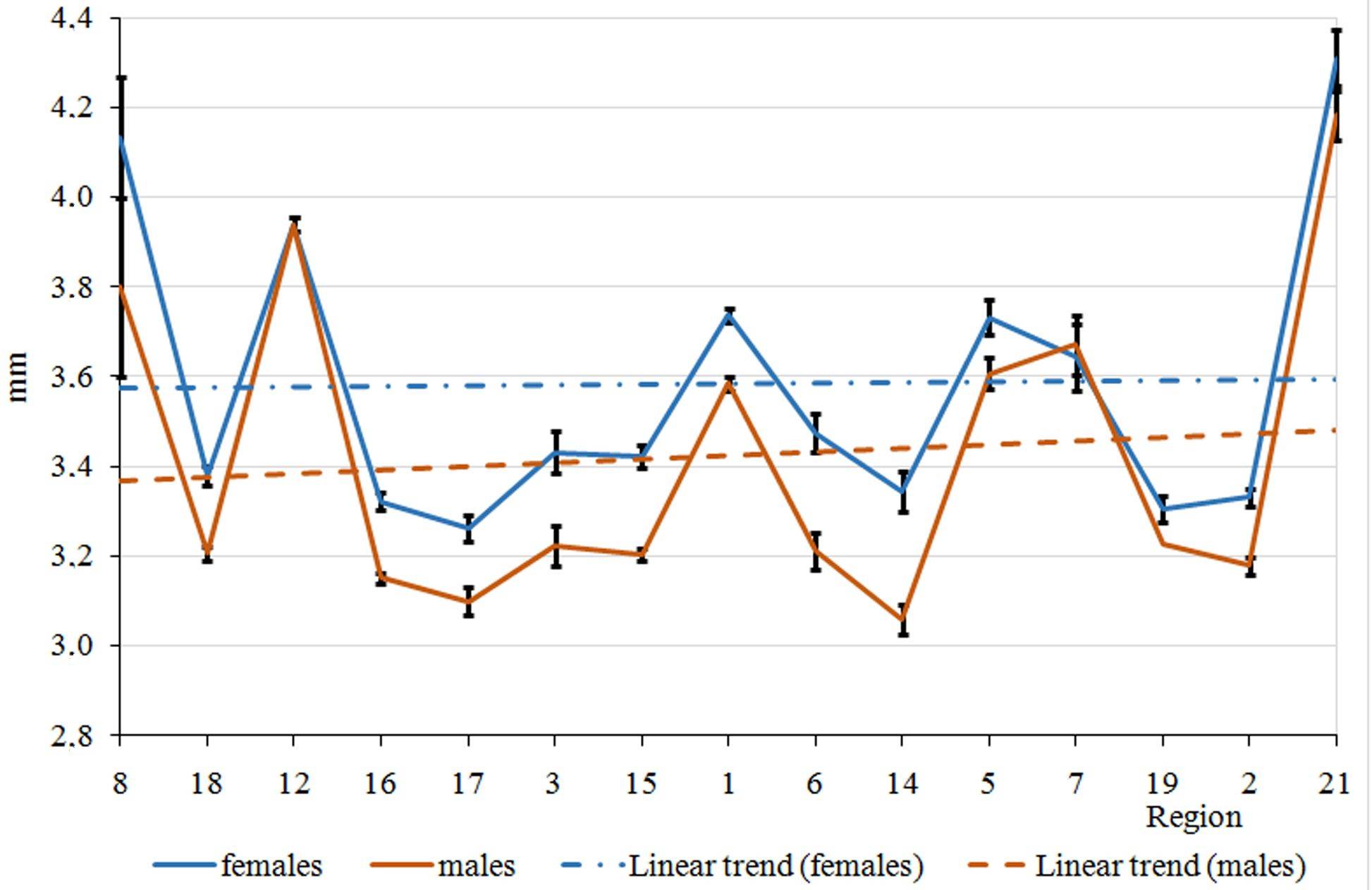

Supplement: Supplementary file 1 [file life-12-00112-s001.zip › S 3.jpg]

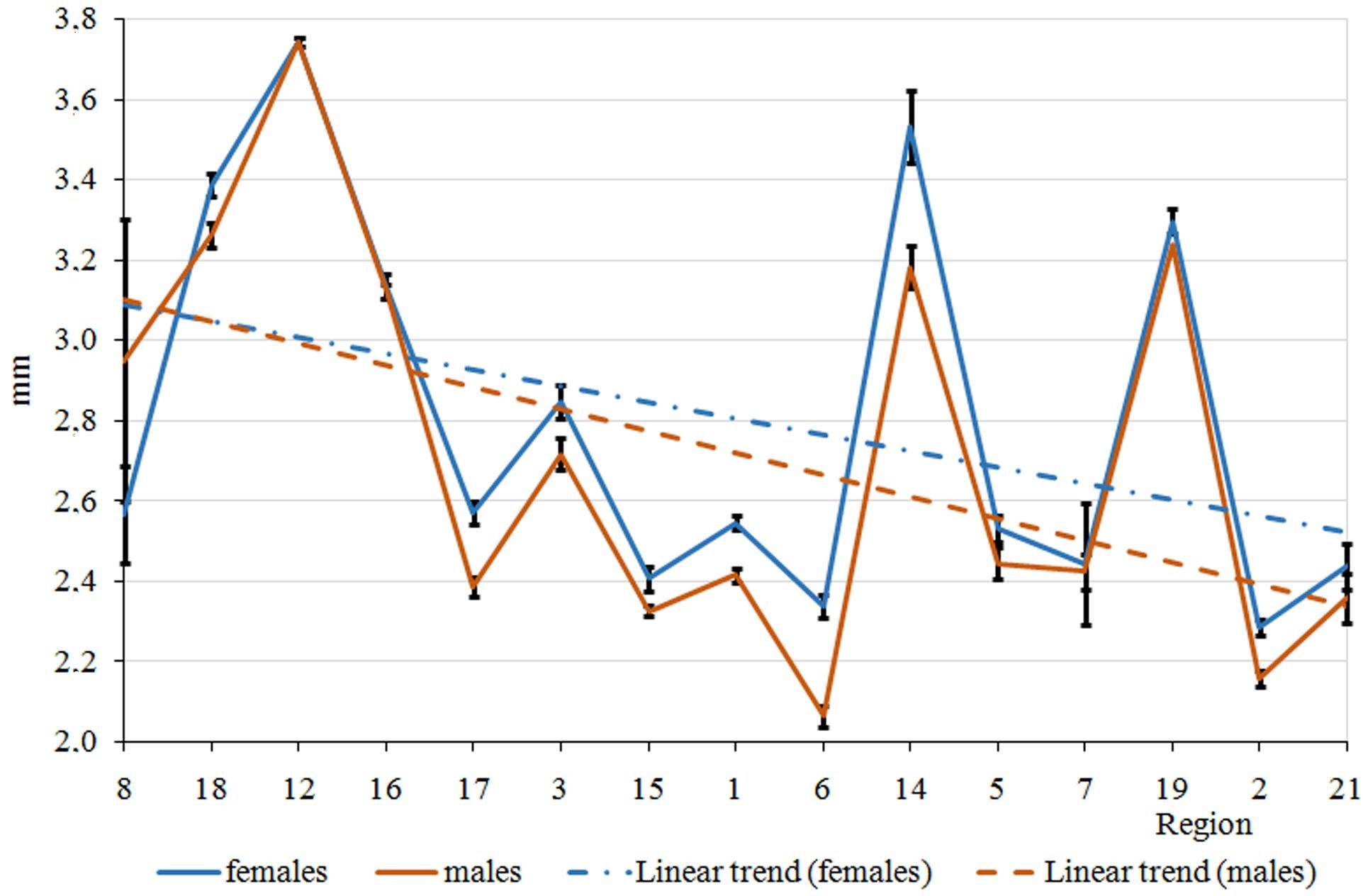

Supplement: Supplementary file 1 [file life-12-00112-s001.zip › S 4.jpg]

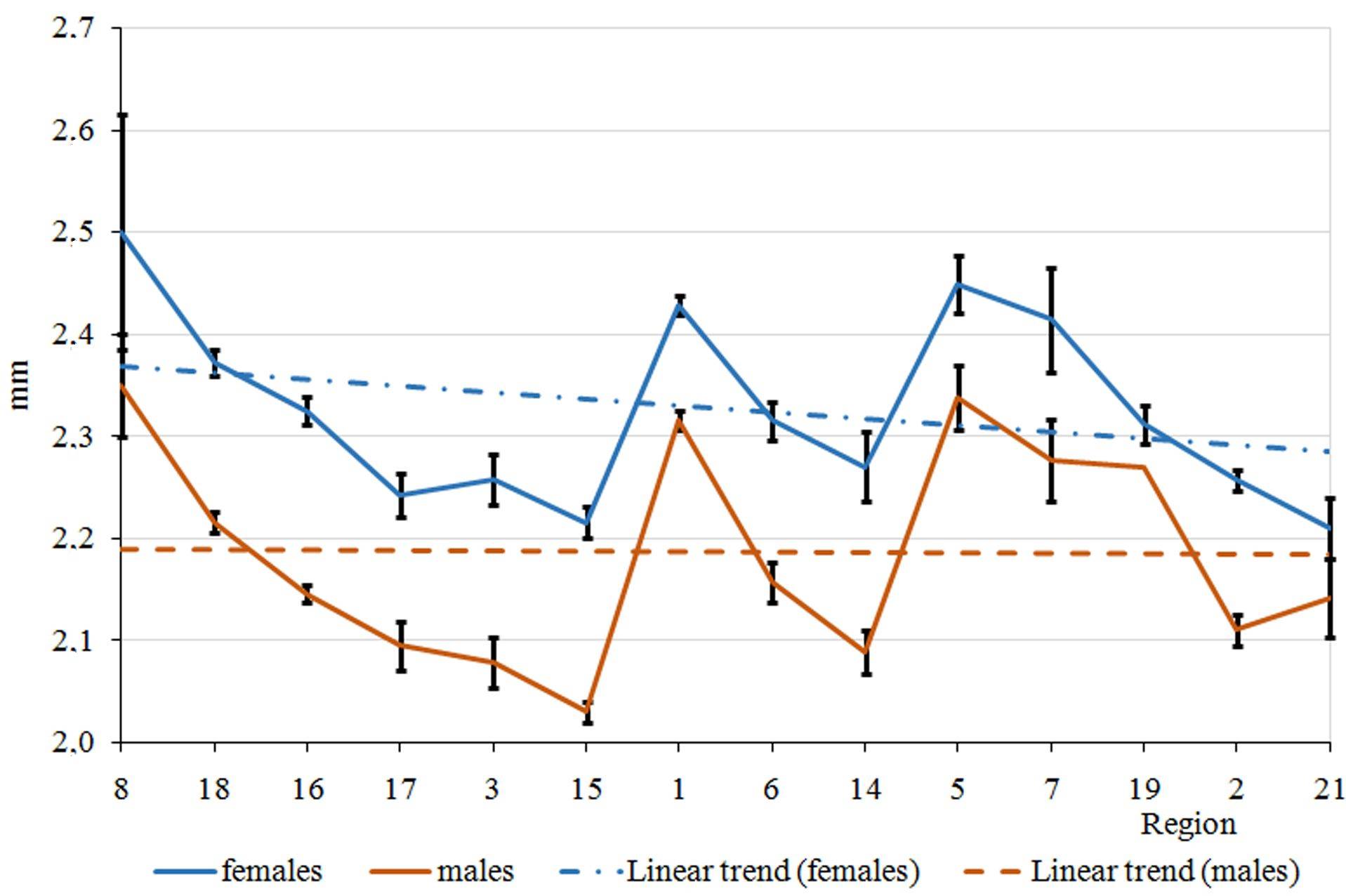

Supplement: Supplementary file 1 [file life-12-00112-s001.zip › S 5.jpg]

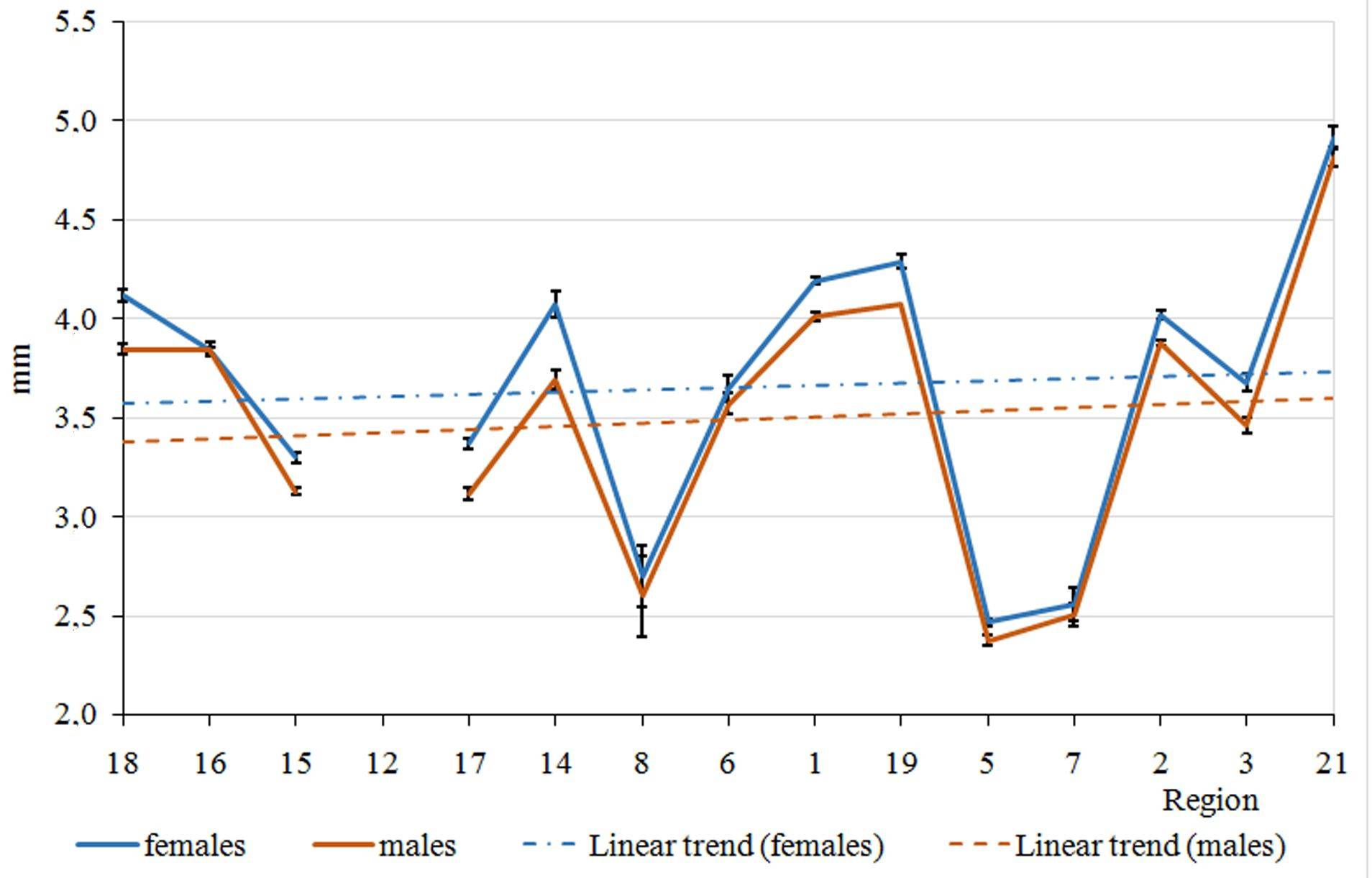

Supplement: Supplementary file 1 [file life-12-00112-s001.zip › S 6.jpg]

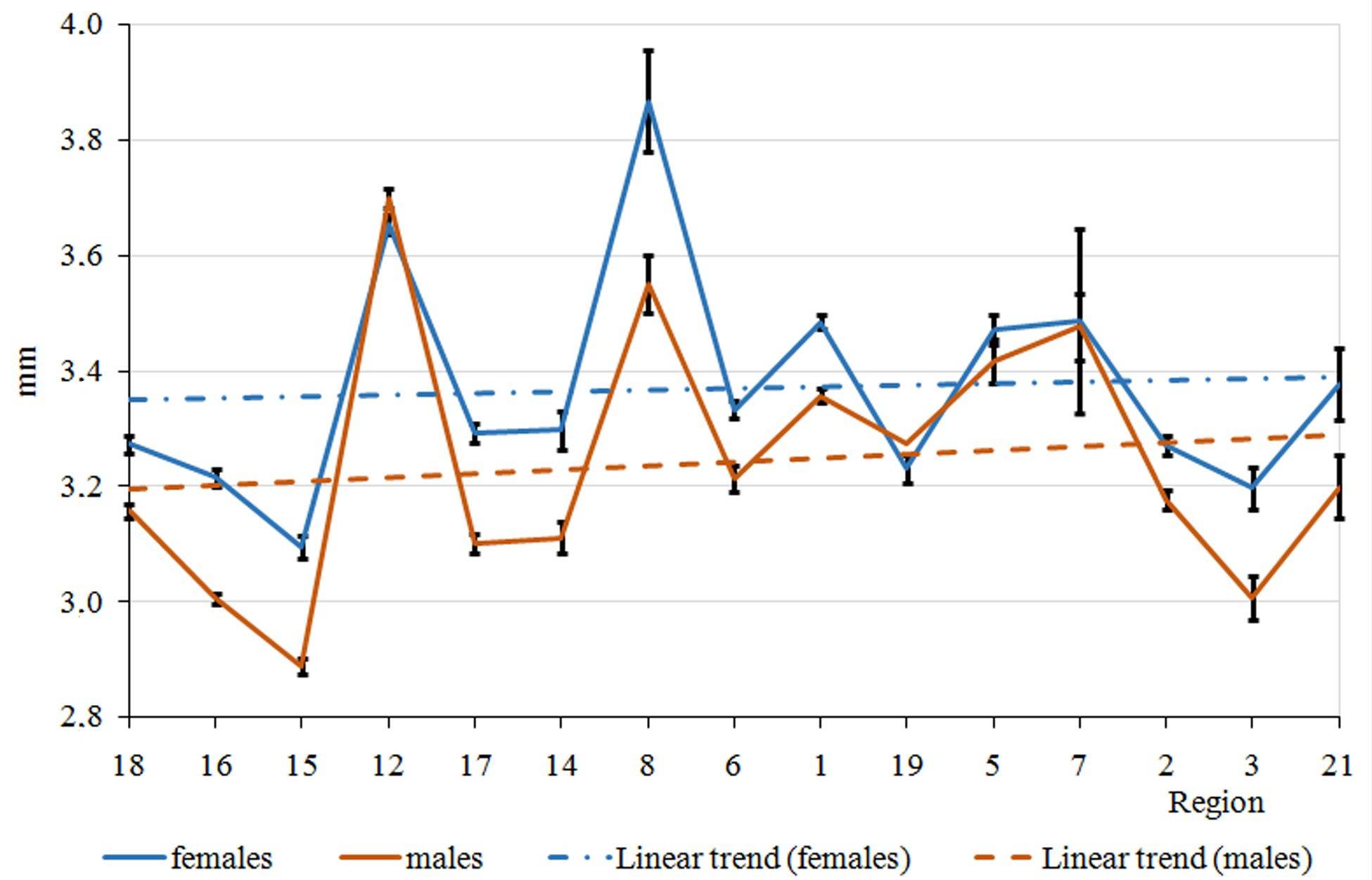

Supplement: Supplementary file 1 [file life-12-00112-s001.zip › S 7.jpg]

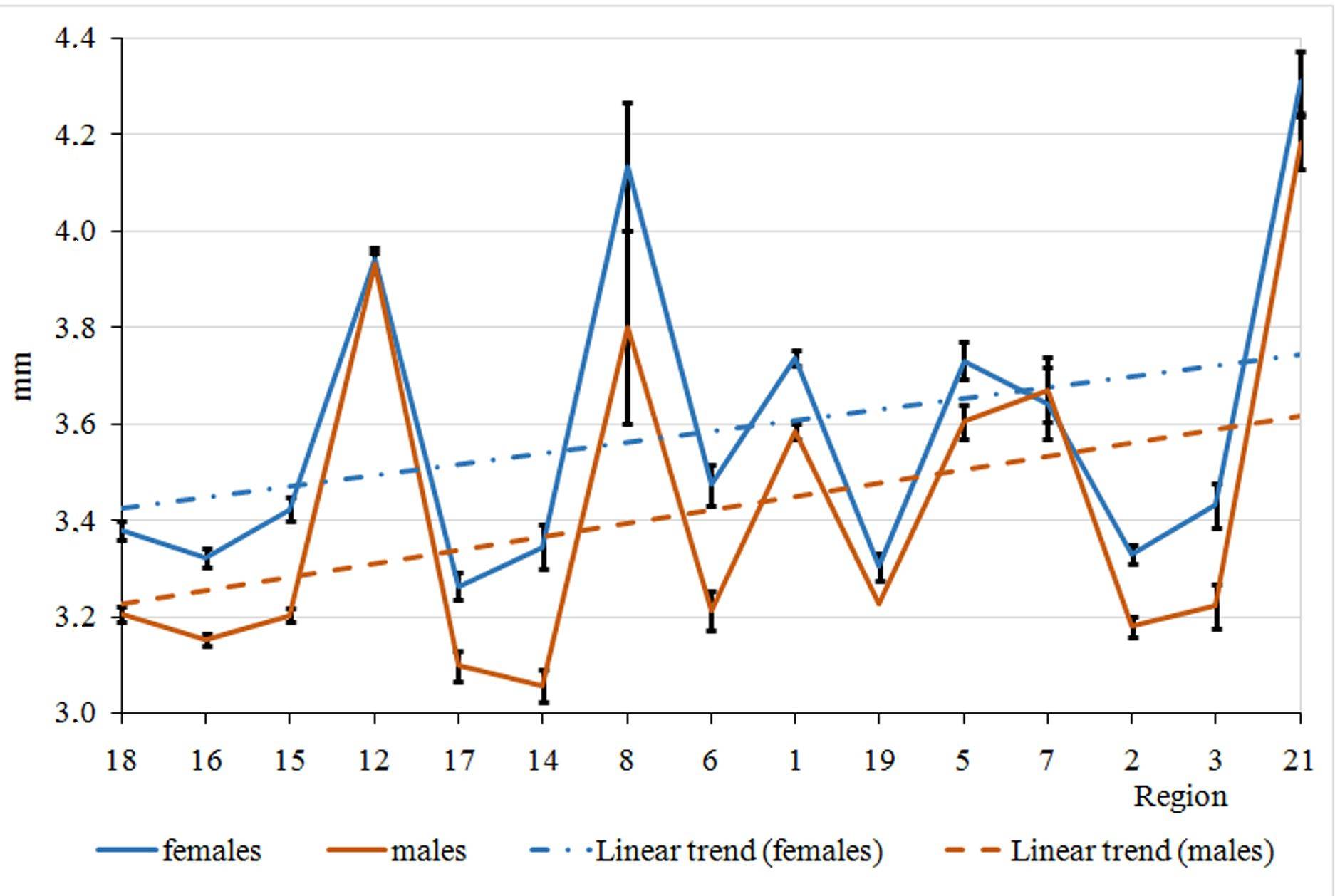

Supplement: Supplementary file 1 [file life-12-00112-s001.zip › S 8.jpg]

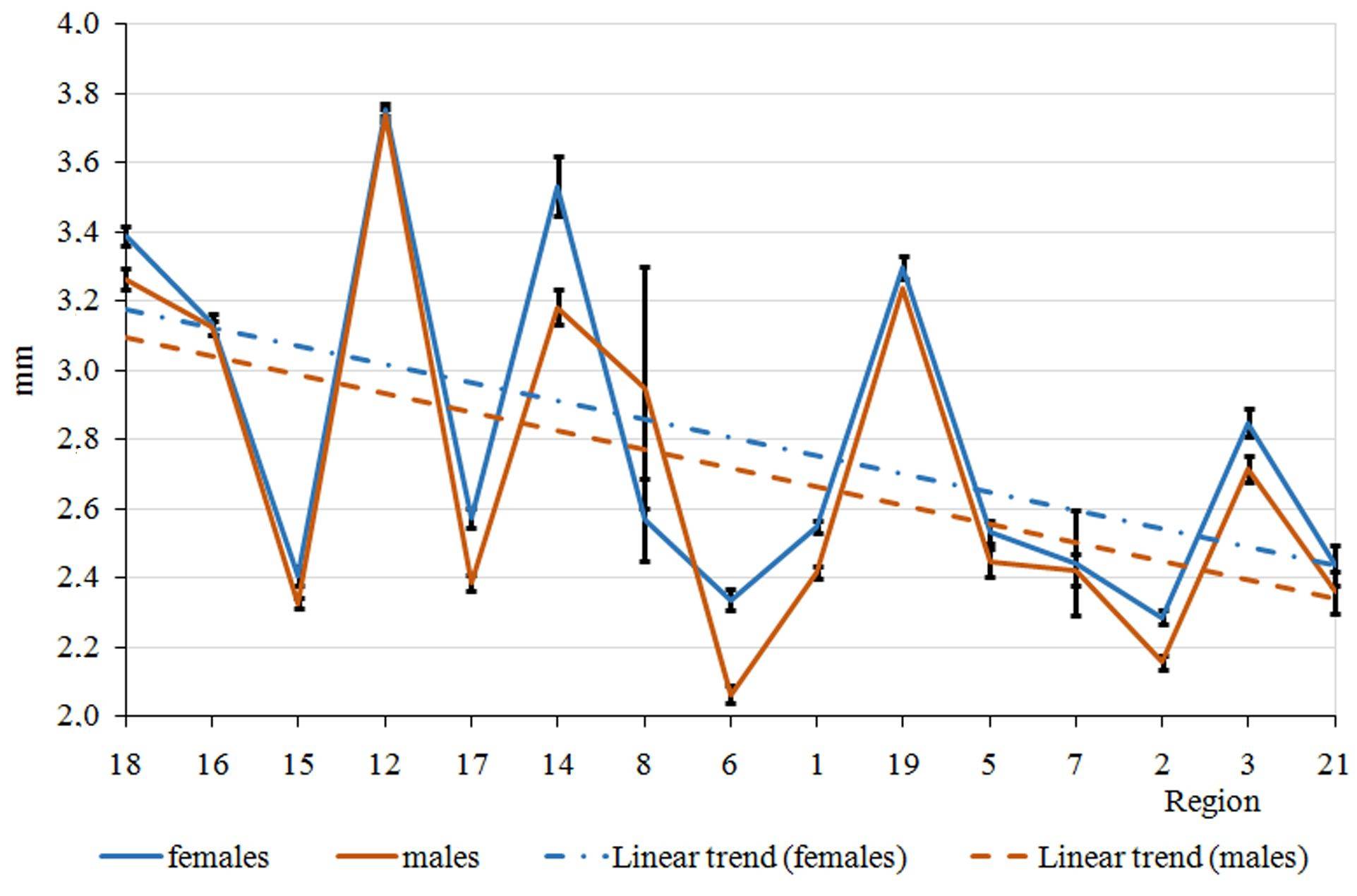

Supplement: Supplementary file 1 [file life-12-00112-s001.zip › S 9.jpg]
